# Does Minimally-Invasive Pancreaticoduodenectomy Have Advantages over Its Open Method? A Meta-Analysis of Retrospective Studies

**DOI:** 10.1371/journal.pone.0104274

**Published:** 2014-08-13

**Authors:** Han Qin, Jianguo Qiu, Yiyang Zhao, Gang Pan, Yong Zeng

**Affiliations:** 1 Department of Hepatic Surgery, West China Hospital, Sichuan University, Chengdu, Sichuan, P. R. China; 2 Department of Hepatobiliary Surgery, The First Affiliated Hospital, Chongqing Medical University, Chongqing, P. R. China; 3 Department of Pediatric Surgery, West China Hospital, Sichuan University, Chengdu, Sichuan, P. R. China; Technische Universität München, Germany

## Abstract

**Background:**

While more and more open procedures now routinely performed using laparoscopy, minimally invasive pancreaticoduodenectomy (MIPD) remains one of the most challenging abdominal procedures. Therefore, we carried out this meta-analysis to evaluate whether MIPD is safe, feasible and worthwhile.

**Methods:**

PubMed, EMBASE, and Cochrane Library were searched to identify studies published between January 1994 and November 2013 comparing MIPD with open pancreaticoduodenectomy (OPD). Intraoperative outcomes, oncologic safety, postoperative complications, and postoperative recovery were evaluated.

**Results:**

11 retrospective studies representing 869 patients (327 MIPDs, 542 OPDs) were included. MIPD was associated with a reduction in estimated blood loss (MD −361.93 ml, 95% CI −519.22 to −204.63 ml, p<0.001, I^2^ = 94%), wound infection (OR 0.41, 95% CI 0.22 to 0.78, p = 0.007, I^2^ = 0%), and hospital stay (MD −2.64 d, 95% CI −4.23 to −1.05 d, p = 0.001, I^2^ = 78%). However, it brings longer operative time (MD 105 min, 95% CI 49.73 to 160.26 min, p<0.001, I^2^ = 93%). There were no significant differences between the two procedures in likelihood of overall complications (p = 0.05), pancreatic fistula (PF) (p = 0.86), delayed gastric empting (DGE) (p = 0.96), positive surgical margins (p = 0.07), retrieval of lymph nodes (p = 0.48), reoperation (p = 0.16) and mortality (p = 0.64).

**Conclusions:**

Our results suggest that MIPD is currently safe, feasible and worthwhile. But considering the selection bias, complexity of MIPD and lack of long-term oncologic outcomes, we suggest it be performed in a high-volume pancreatic surgery center in selected patients.

## Introduction

Laparoscopy has become widely accepted in many intra-abdominal surgeries and has proved to be beneficial to patients in terms of postoperative recovery, reduction of complications and hospital stay [1]–[5] on various occasions. And laparoscopic cholecystectomy has even become the gold standard for surgical removal of the gallbladder. Even though, MIPD is still not universally practiced, because the complexity of the procedure has questioned the safety and advantages over its open counterpart. With the maturation of surgeons' laparoscopic skills and advances in technology, including surgical robotics, MIPD now received more interest.

In recent years, a large number of single-institution series of MIPD have been performed and a variety of studies have been reported [6]–[10]. However, there is currently no powerful evidence that informs the advantages of using laparoscopy over conventional OPD.

The purpose of our study is to critically evaluate whether MIPD, including laparoscopic and robot-assisted laparoscopic, is safe, feasible and worthwhile. Therefore, we carried out a systematic review of the literatures and a meta-analysis of MIPD vs OPD to evaluate the intraoperative outcomes, postoperative complications, postoperative recovery, and oncologic safety. To our knowledge, this meta-analysis is the first to expound this important issue in different subgroups.

## Methods

A systematic review was conducted according to a prespecified protocol based on guidance from the Centre for Reviews and Dissemination [11] and the Cochrane Handbook [12]. The review is reported on the basis of the PRISMA (Preferred Reporting Items for Systematic Reviews and Meta-Analyses) guidelines [13]. We defined MIDP as: using laparoscopy or robot to complete resection of the head of pancreas and duodenum, as well as reconstruction of digestive duct's continuity.

### Eligibility Criteria

Eligibility criteria for all included studies were: (1) meeting the definition of MIPD; (2) comparing MIPD with OPD [acceptable study designs were prospective controlled trials (CTs), cohort studies, and case control studies]; (3) reporting at least one of the outcomes of interest, such as: intraoperative outcomes, oncologic safety, postoperative complications, and postoperative recoveries.

No limits were placed on publication status or language. Translators were consulted as necessary. We excluded studies with any of the following: (1) nonhuman subjects; (2) it was impossible to extract appropriate data from the published articles; (3) there was considerable overlap between authors, institutes, or patients in the published literatures.

### Search Strategy

In accordance with the prespecified study protocol, PubMed, EMBASE, and Cochrane Library were systematically searched with the assistance of 2 independent reviewers (Gang Pan and Yiyang Zhao) from January 1, 1994 (the year MIPD first reported) to November 17, 2013(the day we completed the literature search). The prespecified search terms were grouped in 3 areas: the “minimally invasive” terms (minimally invasive/laparoscopic/laparoscopy/robotic/Da Vinci/robotic-assisted/laparoscopic-assisted), the “open” terms (open/conventional), and the “pancreaticoduodenectomy” terms (pancreaticoduodenectomy/whipple/duodenopancreatectomy/pancreatectomy/pancreatic).

### Study Selection

The study selection process took place in 2 consecutive steps. In Phase 1, a manual selection of the potentially relevant articles was performed by scanning their title and abstract. In Phase 2, the full-text versions of articles selected in Phase 1 were assessed. Two reviewers (Qiang Liu and Jinhai Gou) independently evaluated all retrieved articles using prespecified eligibility criteria. In case of disagreement, a consensual decision was made.

### Data Extraction

Two reviewers (Gang Pan and Yiyang Zhao) extracted data from all selected studies in RevMan 5.0 software independently. The same consensus process mentioned above was used to resolve disagreements. The data extracted included year of study publication, study country, study type, patient demographics, definitions of PF&DGE, conversion rate, intraoperative outcomes, oncologic safety, postoperative complications, and postoperative recovery. If possible, the first or corresponding author was contacted to obtain supplementary information when there were missing data or inaccuracy in the information. If the author failed to respond, the study was excluded from the outcome analysis.

### Quality Assessment

The quality of the studies was assessed by using the Modified Newcastle–Ottawa Score [14], which allocates a maximum of 9 points each to patient selection, the comparability of the two groups (MIPD and OPD), and outcome assessment. Two authors (Han Qin and Jianguo Qiu) examined the studies independently. The same consensus process mentioned above was used to resolve disagreements.

### Statistical Methods

This meta-analysis was performed in line with recommendations from the Cochrane Collaboration [15] and the Quality of Reporting of Meta-analyses [16] guidelines. The statistical software Review Manager version 5.0 (The Cochrane Collaboration, Oxford, United Kingdom) was used to perform all statistical analyses. I^2^ values were used for quantification of statistical inconsistency, defined as the percentage of variation between studies due to heterogeneity [17]. And a value exceeding 50% was considered to represent significant heterogeneity. A random-effects model was used to report the results of heterogeneous data, otherwise a fixed-effects model was used. Continuous variables was conducted with the Inverse-Variance statistical method by using weighted mean difference (MD), and dichotomous variables were analyzed with the Mantel-Haenszel statistical method using odds ratio (OR) as the summary statistic, and both were reported with 95% confidence intervals (CI). Funnel plots were constructed to detect and assess publication bias and any associations between treatment estimates and sample size. Forest plots were constructed, and the value of P<0.05 was considered to indicate statistical significance.

## Results

**Figure 1:**
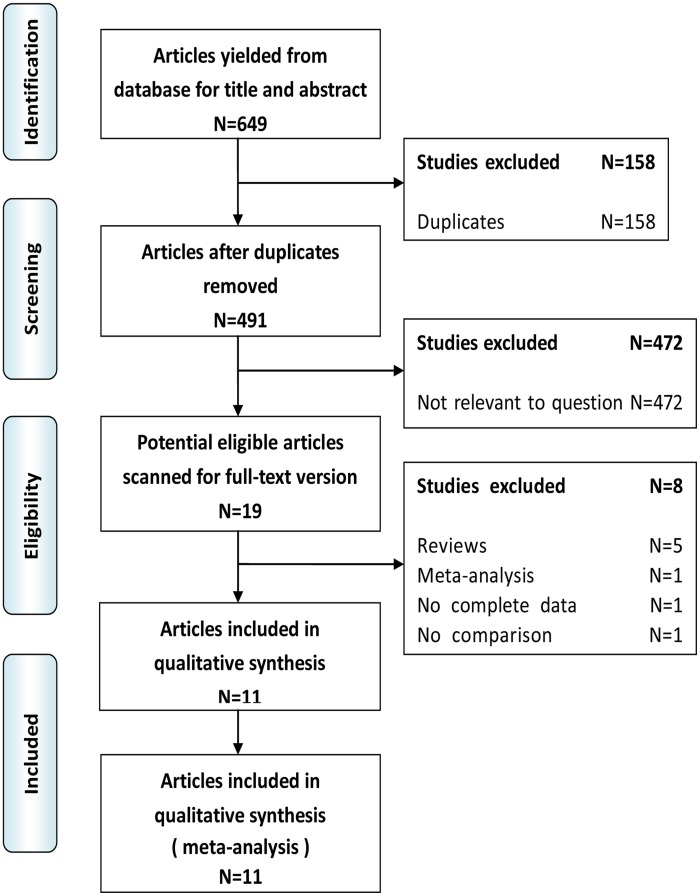
The PRISMA flowchart of literature review.

The literature search yielded 649 studies initially. No other eligible studies were found from other sources. In phase 1 of the study selection process, 19 potential eligible articles were included for a full-text version after screening their titles and abstracts. Of these, we excluded 5 [18]–[22], because they were reviews of MIPD. Nakamura M et al [23] was excluded for it was a meta-analysis of laparoscopic pancreatic resection vs open pancreatic resection. Gumbs AA et al [24] was excluded because the data was not extractable and the authors could not be reached to provide additional information. Another study [25] in which no comparison was found was also excluded. Finally, this left a total of 11 studies [26]–[36] representing 869 patients for inclusion in the meta-analysis. The PRISMA flow chart of literature search strategies is illustrated in **Figure 1** **.**

### Study Characteristics

Characteristics of included studies are presented in **Table 1**. All eleven were retrospective studies of variable quality that compared consecutive cases of MIPD with either consecutive or matched OPD performed during the same period. They were varied with respect to age, gender, body mass index (BMI), American Society of Anesthesiologists (ASA) score, and operation indication. For example, Buchs NC et al [36] reported that the ASA score was 2.5±0.5 in the MIPD group, while it was 2.15±0.7 in the OPD group (p = 0.01). And the percentage of patients with pancreatitis was 3.36% in the MIPD group, while it was 6.83% in the OPD group. Most of the referred studies used the definitions from ISGPF&ISGPS [37], [38] to define pancreatic fistula and delayed gastric emptying, but some might use the others like the definition from Suc B et al [39]. All the conversions were included in the MIPD group. The majority of cases were performed for malignancy, but no report of long-term oncologic result. In most cases, the selection criteria for patients to undergo MIPD were not reported.

**Table 1:** Characteristics of included studies.

Study	Year	Country	No. Patients		Mean Age(Yrs)		Male(%)		Definition of PF	Definition of DGE	Conversion rate, (%)	Quality scores	Type of MIPD	Operation indications
			MIPD	OPD	MIPD	OPD	MIPD	OPD						
Kuroki T et al.^26^	2012	Japan	20	31	71.2±8.8	73.5±7.3	55	68	ISGPF	ISGPS	0	6	NRA	1,2,3,4,5,7
Zureikat AH et al.^27^	2011	USA	14	14	79.8±10.2	67.4±11	79	50	ISGPF	NR	14	4	NRA	1,2,3,4,6,7,8
Cho A et al.^28^	2009	Japan	15	15	64±10	68±9	40	47	ISGPF	ISGPS	0	6	NRA	1,2,3,4,5,8,9
Asbun HJ et al.^29^	2012	USA	53	215	62.9±14.14	67.3±11.53	55	44	ISGPF	ISGPS	15	8	NRA	1,2,3,4,5,7,8,9
Mesleh MG et al.^30^	2013	USA	75	48	NR	NR	57	48	ISGPF	ISGPS	13	4	NRA	3,4,9
Li Y et al.^31^	2013	China	20	47	57±11	58±10	60	68	NR	ISGPS	0	3	NRA	1,2,3,6,7
Chalikonda S et al.^32^	2012	USA	30	30	62±11.4	61±12.7	54	54	ISGPF	ISGPS	10	6	RA	1,2,3,4
Zhou NX et al.^33^	2011	China	8	8	64.38±9.08	59.38±9.38	63	50	Suc B et al.	NR	0	5	RA	1,2,3,8,9
Bao PQ et al.^34^	2013	USA	28	28	68.0±11.2	67.7±12.5	46	46	ISGPF	ISGPS	14	8	RA	1,2,3,4,5,6,7,8
Lai EC et al.^35^	2012	Hong Kong	20	67	66.4±11.9	62.1±11.2	60	57	NR	NR	5	6	RA	1,2,3,4,5,6,7,8,9
Buchs NC et al.^36^	2011	USA	44	39	63±14.5	56±15.8	50	36	ISGPF	NR	4.5	7	RA	1,2,3,4,5,7,8,9

NR, no record; NRA, no robot-assisted; RA, robot-assisted; Operation indications: 1.Cholangiocarcinoma 2.Pancreatic adenocarcinoma 3.Periampullar adenocarcinoma 4.Intraductal pancreatic mucinous neoplasm 5.pancreatic neuroendocrine tumor 6.gastrointestinal stromal tumor 7.Pancreatitis 8.Periampullar adenoma 9.Else.

### Quality Assessment

Evaluation of methodological quality based on the Cochrane risk of bias tool was not performed, because there was no RCT. However, we used the Modified Newcastle–Ottawa Score instead. The quality of the studies was evaluated by examining three factors: patient selection, comparability of the study groups and assessment of outcomes. A score of 0–9 was allocated to each study. In general, 7 studies were considered to be of high quality by achieving a score of ≥6. The scores of those studies are also presented in **Table 1**. Results from the meta-analysis with regard to intraoperative outcomes, postoperative outcomes, and oncologic safety are summarized in **Table 2**.

**Table 2:** Comparison of MIPD Versus OPD (Pooled Analysis).

Outcome of interest	Studies, n	Patients, n	OR/MD	95% CI	P value	Heterogeneity Test	
						P value	I^2^
**Intraoperative outcomes**							
Operative time,min	11	869	105	(49.73, 160.26)	**<0.001**	<0.001	93%
Estimated blood loss, mL	10	746	−361.93	(−519.22 −204.63)	**<0.001**	<0.001	94%
**Postoperative outcomes**							
Morbidity, (%)	9	762	0.73	(0.53, 1.00)	0.05	0.35	10%
Wound infection, n	6	584	0.41	(0.22, 0.78)	**0.007**	0.86	0%
Overall pancreatic fistula, n	10	802	0.96	(0.65, 1.44)	0.86	0.88	0%
Grade A	5	486	0.88	(0.47, 1.66)	0.7	0.86	0%
Grade B	6	548	1.01	(0.46, 2.20)	0.98	0.89	0%
Grade C	6	525	0.83	(0.37, 1.85)	0.64	0.87	0%
Overall delayed gastric emptying, n	11	869	0.99	(0.62, 1.56)	0.96	0.76	0%
Grade A	3	386	0.82	(0.31, 2.19)	0.69	0.49	0%
Grade B	4	446	0.77	(0.29, 2.02)	0.59	0.93	0%
Grade C	3	365	1.23	(0.41, 3.71)	0.71	0.8	0%
Reoperation, n	8	721	0.63	(0.34, 1.19)	0.16	0.73	0%
Mortality, (%)	7	582	0.82	(0.37, 1.85)	0.64	0.93	0%
Length of stay, d	10	818	−2.64	(−4.23, −1.05)	**0.001**	<0.001	78%
**Oncologic outcomes**							
Retrieved lymph nodes, n	7	612	1.15	(−2.02, 4.32)	0.48	<0.001	83%
Positive surgical margins,n	7	451	0.57	(0.31, 1.04)	0.07	0.12	40%

## Pooled Analysis

### Intraoperative Outcomes

All the eleven studies reported data on operative time, and when a random-effects model was used, the meta-analysis showed that the use of minimally invasive procedures brings longer operative time (MD 105 min, 95% CI 49.73 to 160.26 min, p<0.001, I^2^ = 93%). Estimated blood loss was reported in ten studies^26∼29, 31∼36^ which included 746 patients. The meta-analysis showed that MIPD gives a reduction in intraoperative estimated blood loss (MD −361.93 ml, 95% CI −519.22 to −204.63 ml, p<0.001, I^2^ = 94%)

### Postoperative Outcomes

**Figure 2:**
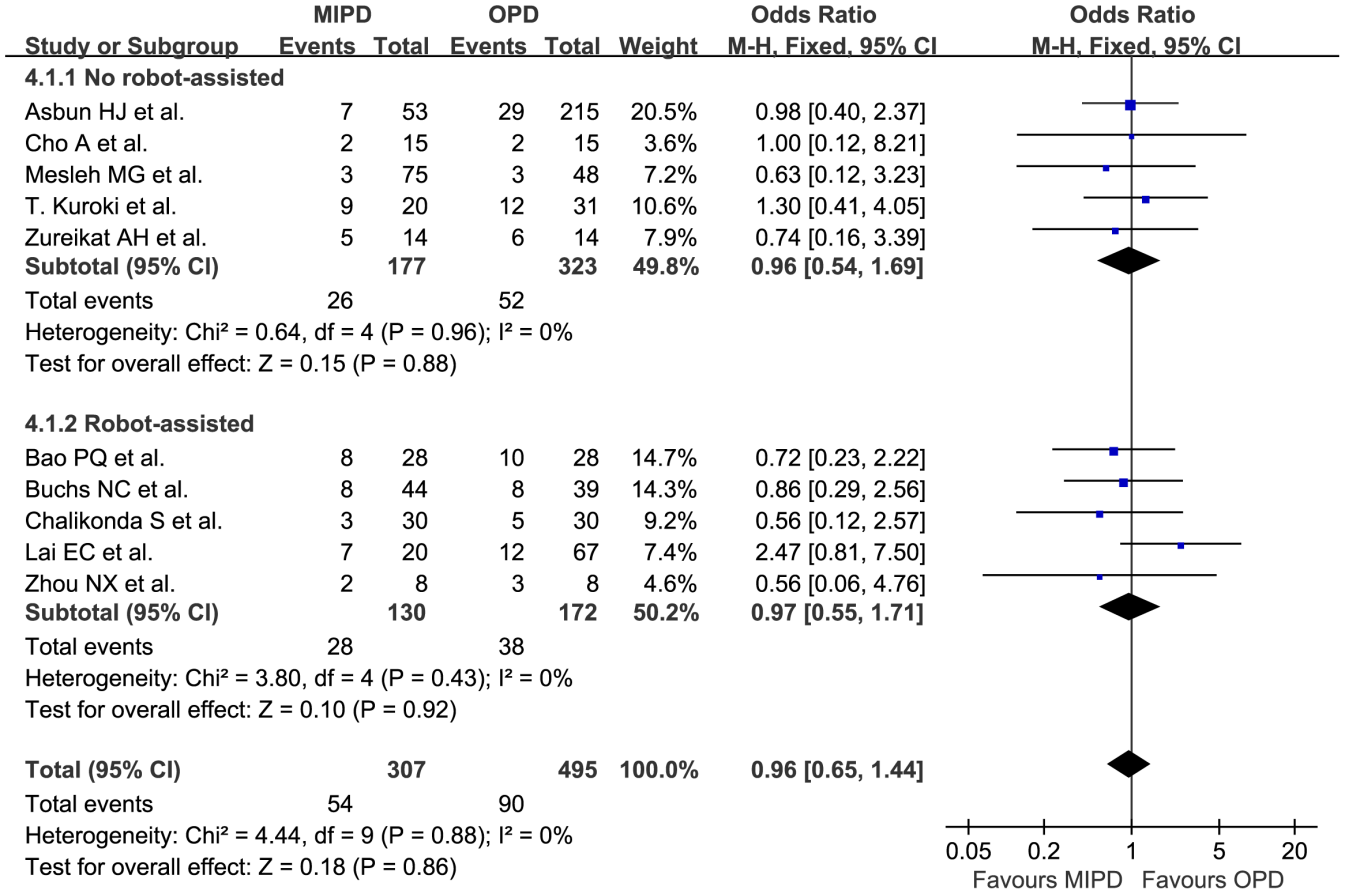
Pooled meta-analysis of pancreatic fistula, comparing MIPD with OPD.

**Figure 3:**
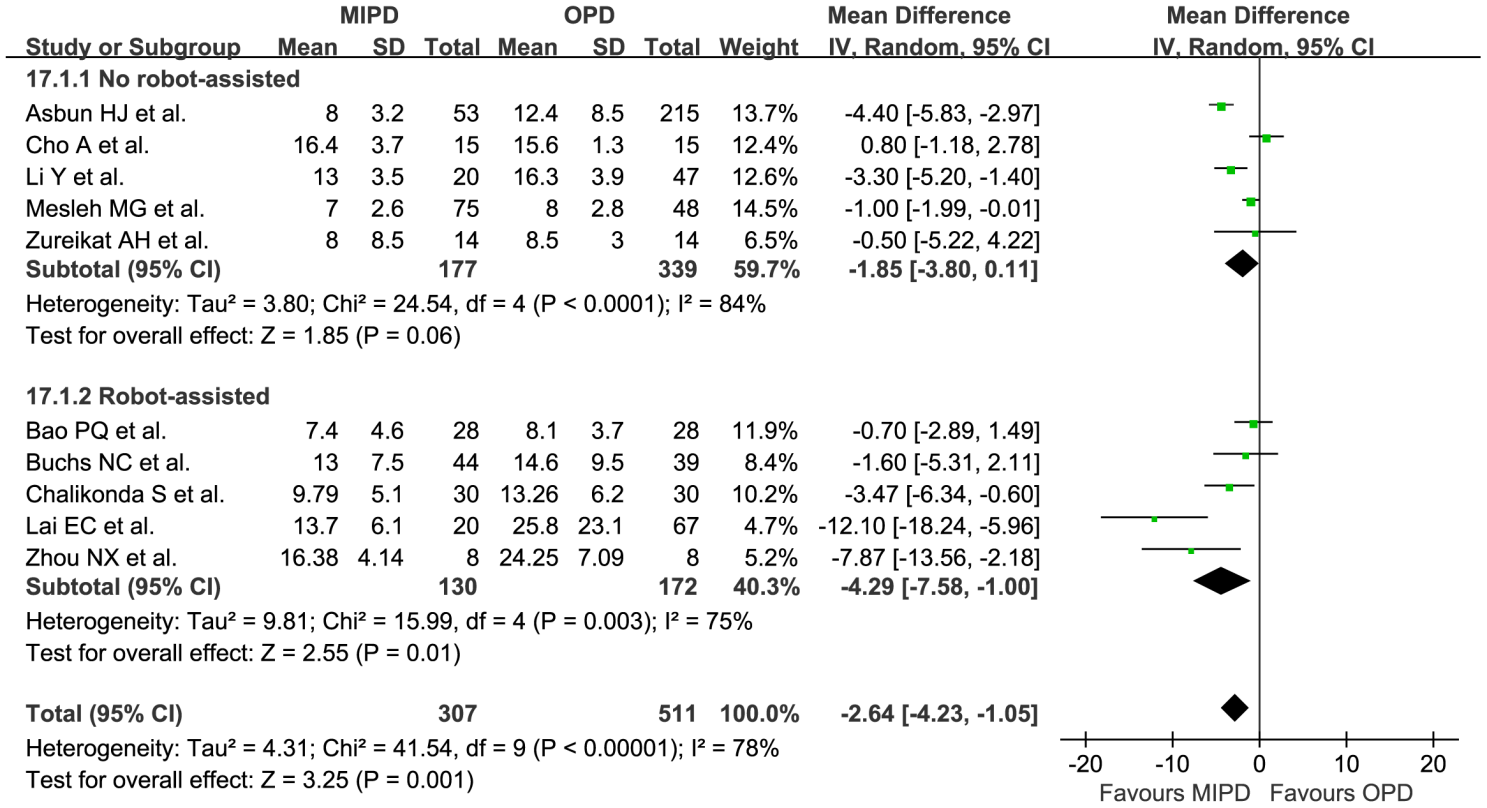
Pooled meta-analysis of length of stay, comparing MIPD with OPD.

Nine studies^27∼33, 34, 35^ representing 746 patients reported overall complications, and the meta-analysis showed there was no statistical difference between MIPD and OPD (OR 0.73, 95% CI 0.53 to 1.00, p = 0.05, I^2^ = 10%). Six studies^28, 29, 32, 34∼36^ showed that MIPD has fewer wound infections (OR 0.41, 95% CI 0.22 to 0.78, p = 0.007, I^2^ = 0%). The meta-analysis of ten studies including 802 patients indicated that there was no significant statistical difference between MIPD and OPD in the incidence of neither overall pancreatic fistula (OR 0.96, 95% CI 0.65 to 1.44, p = 0.86, I^2^ = 0%) (Fig. 2) nor overall delayed gastric emptying (OR 0.99, 95% CI 0.62 to 1.56, p = 0.96, I^2^ = 0%). Besides, when dividing PF&DGE into 3 subgroups of Grade A, B, and C, according to the guidelines of ISGPF& ISGPS^37, 38^ respectively, there was still no statistical difference between MIPD and OPD. Eight studies^27, 29, 30, 32∼36^ representing 721 patients reported the incidence of reoperation, the meta-analysis showed there was no statistical difference between the two procedures (OR 0.63, 95% CI 0.34 to 1.19, p = 0.16, I^2^ = 0%). The meta-analysis of seven studies^27, 29, 31∼35^ including 582 patients showed there was no statistical difference in mortality (OR 0.82, 95% CI 0.37 to 1.85, p = 0.64, I^2^ = 0%). Length of stay was shorter by 2.64 days for the MIPD group, and the difference was statistically significant (MD −2.64 d, 95% CI −4.23 to −1.05 d, p = 0.001, I^2^ = 78%) (**Fig. 3**).

### Oncologic Outcomes

**Figure 4:**
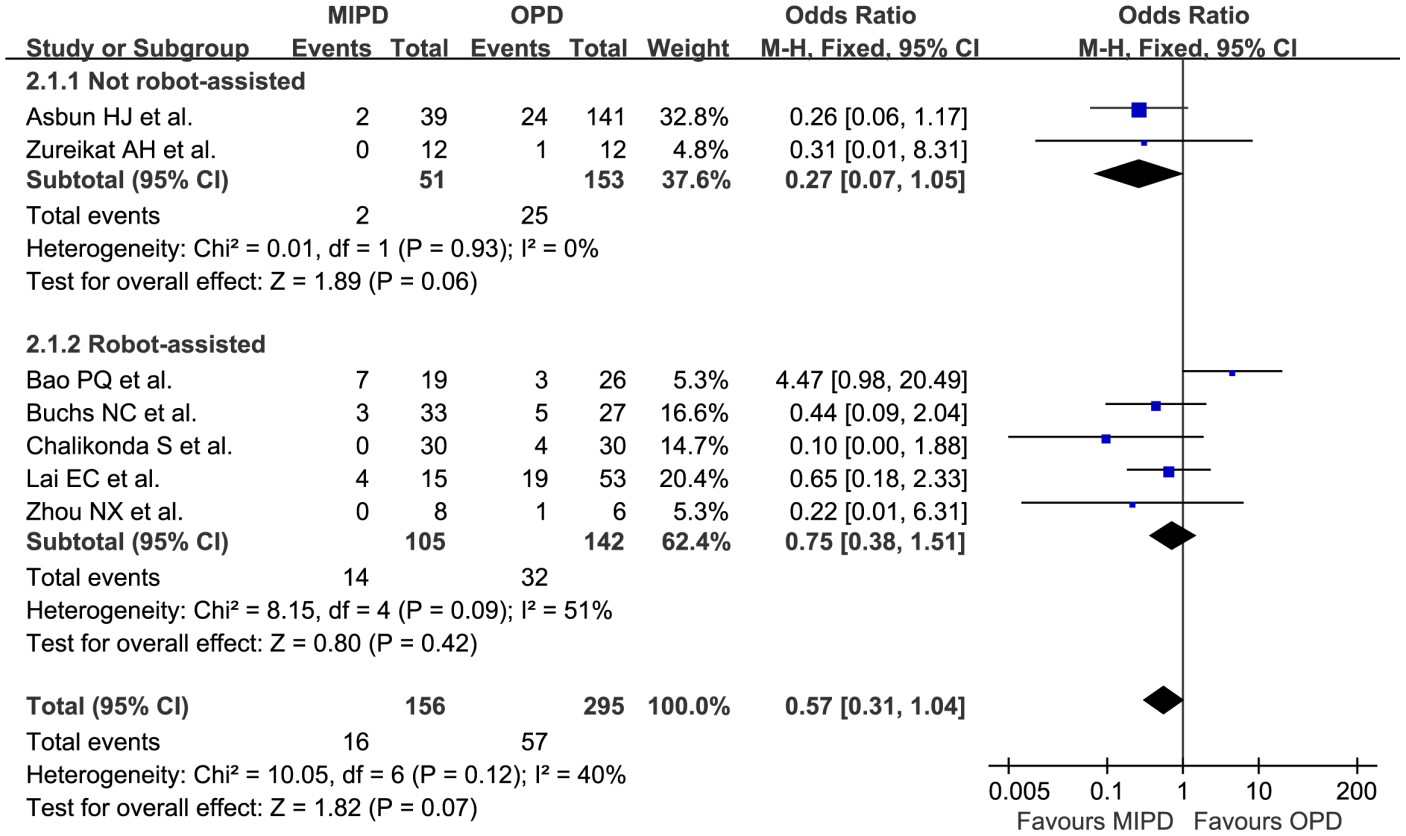
Pooled meta-analysis of positive surgical margins, comparing MIPD with OPD.

The number of retrieved lymph nodes was mentioned in 7 studies^27∼29, 32, 34∼36^, representing 612 patients. The meta-analysis showed there was no significant statistical difference between MIPD and OPD (MD 1.15, 95% CI −2.02 to 4.32, p = 0.48, I^2^ = 83%). As to the rate of positive surgical margins, which mean they were not R0 resections, the meta-analysis showed there was no statistical difference (OR 0.57, 95% CI 0.31 to 1.04, p = 0.07, I^2^ = 40%). (**Fig. 4**)

### Sensitivity Analysis

The sensitivity analysis included the following: (1) NRA: 6 studies which did not use robot; (2) RA: 5 studies which used robot; (3) HQ: 7 studies of high quality (with quality score of 6 or more using the modified Newcastle-Ottawa scale); and (4) large sample size (LSS): 4 studies with at least 30 patients in each procedure. Results from sensitivity analysis were summarized in **Table 3**.

**Table 3:** Comparison of MIPD Versus OPD (Sensitivity Analysis).

Outcome of interest	Studies, n	Patients, n	OR/MD	95% CI	P value	Heterogeneity Test	
						P value	I^2^
**Analysis of NRA subgroup**							
* * ***Intraoperative outcomes***							
Operative time,min	6	567	119.46	(77.61, 161.30)	**<0.001**	<0.001	82%
Estimated blood loss, mL	5	444	−451.62	(−867.47, −35.77)	**0.03**	<0.001	96%
*** Postoperative outcomes***							
Morbidity, (%)	5	516	0.8	(0.53, 1.19)	0.27	0.27	22%
Wound infection, n	2	298	0.47	(0.19, 1.13)	0.09	0.8	0%
Overall pancreatic fistula, n	5	500	0.96	(0.54, 1.69)	0.88	0.96	0%
Grade A	3	347	1.09	(0.50, 2.35)	0.83	0.84	0%
Grade B	3	349	1.06	(0.35, 3.18)	0.92	0.82	0%
Grade C	3	326	0.83	(0.26, 2.61)	0.75	0.6	0%
Overall delayed gastric emptying, n	6	567	1.11	(0.65, 1.90)	0.7	0.51	0%
Grade A	3	386	0.82	(0.31, 2.19)	0.69	0.49	0%
Grade B	3	386	0.74	(0.26, 2.09)	0.57	0.81	0%
Grade C	3	365	1.23	(0.41, 3.71)	0.71	0.8	0%
Reoperation, n	3	419	0.79	(0.27, 2.32)	0.67	0.63	0%
Mortality, (%)	3	363	0.77	(0.27, 2.21)	0.63	0.66	0%
Length of stay, d	5	516	−1.85	(−3.80, 0.11)	0.06	<0.001	84%
*** Oncologic outcomes***							
Retrieved lymph nodes, n	3	326	1.86	(−3.86, 7.59)	0.52	0.009	79%
Positive surgical margins,n	2	204	0.27	(0.07, 1.05)	0.06	0.93	0%
**Analysis of RA subgroup**							
*** Intraoperative outcomes***							
Operative time,min	5	302	100.71	(−35.12, 236.54)	0.15	<0.001	96%
Estimated blood loss, mL	5	444	−287.51	(−454.84, −120.18)	**<0.001**	<0.001	91%
*** Postoperative outcomes***							
Morbidity, (%)	4	246	0.62	(0.36, 1.06)	0.08	0.35	8%
Wound infection, n	4	286	0.35	(0.14, 0.90)	**0.03**	0.64	0%
Overall pancreatic fistula, n	5	302	0.97	(0.55, 1.71)	0.92	0.43	0%
Grade A	2	139	0.58	(0.20, 1.74)	0.34	0.74	0%
Grade B	3	199	0.96	(0.32, 2.89)	0.95	0.54	0%
Grade C	3	199	0.82	(0.27, 2.55)	0.73	0.67	0%
Overall delayed gastric emptying, n	5	302	0.71	(0.28, 1.76)	0.45	0.73	0%
Grade A	0	0	/	/	/	/	/
Grade B	1	60	/	/	/	/	/
Grade C	0	0	/	/	/	/	/
Reoperation, n	5	302	0.56	(0.26, 1.22)	0.15	0.51	0%
Mortality, (%)	4	219	0.92	(0.25, 3.30)	0.89	0.79	0%
Length of stay, d	5	302	−4.29	(−7.58, −1.00)	**0.01**	0.003	75%
*** Oncologic outcomes***							
Retrieved lymph nodes, n	4	286	1.15	(−3.32, 4.59)	0.75	<0.001	85%
Positive surgical margins,n	5	247	0.75	(0.38, 1.51)	0.42	0.09	51%
**Analysis of HQ subgroup**							
*** Intraoperative outcomes***							
Operative time,min	7	635	77.03	(−5.14, 159.21)	0.07	<0.001	95%
Estimated blood loss, mL	7	635	−493.46	(−735.81, −251.12)	**<0.001**	<0.001	92%
*** Postoperative outcomes***							
Morbidity, (%)	5	528	0.62	(0.42, 0.92)	**0.02**	0.76	0%
Wound infection, n	6	584	0.41	(0.22, 0.78)	**0.007**	0.86	0%
Overall pancreatic fistula, n	7	635	1.04	(0.67, 1.61)	0.86	0.72	0%
Grade A	3	402	0.97	(0.46, 2.05)	0.93	0.71	0%
Grade B	6	548	1.01	(0.46, 2.20)	0.98	0.89	0%
Grade C	5	497	0.89	(0.39, 2.05)	0.78	0.84	0%
Overall delayed gastric emptying, n	7	635	0.89	(0.49, 1.63)	0.71	0.89	0%
Grade A	2	319	0.62	(0.16, 2.39)	0.49	0.27	17%
Grade B	3	379	0.94	(0.28, 3.14)	0.92	0.92	0%
Grade C	2	298	1.32	(0.41, 4.29)	0.64	0.56	0%
Reoperation, n	5	554	0.57	(0.28, 1.16)	0.12	0.54	0%
Mortality, (%)	4	471	0.8	(0.32, 2.01)	0.63	0.83	0%
Length of stay, d	6	584	−2.86	(−5.39, −0.33)	**0.03**	<0.001	83%
*** Oncologic outcomes***							
Retrieved lymph nodes, n	6	584	1.38	(−2.13, 4.89)	0.44	<0.001	85%
Positive surgical margins,n	5	413	0.61	(0.32, 1.13)	0.12	0.05	58%
**Analysis of LSS subgroup**							
*** Intraoperative outcomes***							
Operative time,min	4	534	84.06	(−27.20, 195.32)	0.14	<0.001	97%
Estimated blood loss, mL	3	411	−546.47	(−874.87, −218.08)	**0.001**	<0.001	86%
*** Postoperative outcomes***							
Morbidity, (%)	4	534	0.62	(0.42, 0.92)	**0.02**	0.61	0%
Wound infection, n	3	411	0.44	(0.19, 1.00)	0.05	0.57	0%
Overall pancreatic fistula, n	4	534	0.82	(0.45, 1.48)	0.51	0.92	0%
Grade A	2	351	0.77	(0.30, 2.00)	0.6	0.81	0%
Grade B	3	411	1.08	(0.32, 3.69)	0.9	0.57	0%
Grade C	3	411	0.85	(0.31, 2.30)	0.74	0.71	0%
Overall delayed gastric emptying, n	4	534	1.02	(0.53, 1.96)	0.96	0.69	0%
Grade A	1	268	/	/	/	/	/
Grade B	2	328	0.84	(0.22, 3.25)	0.81	0.89	0%
Grade C	1	268	/	/	/	/	/
Reoperation, n	54	534	0.51	(0.23, 1.12)	0.09	0.62	0%
Mortality, (%)	2	328	0.78	(0.26, 2.34)	0.65	0.36	0%
Length of stay, d	4	534	−2.64	(−4.79, −0.49)	**0.02**	0.001	81%
*** Oncologic outcomes***							
Retrieved lymph nodes, n	3	411	4.5	(1.12, 7.89)	**0.009**	0.02	73%
Positive surgical margins,n	3	300	0.27	(0.10, 0.73)	**0.01**	0.65	0%

### NRA subgroup

When only studies without robot were analyzed, it comes out that there was no statistical difference in length of stay (MD −1.85 d, 95% CI, −3.80 to 0.11 d, P = 0.06, I^2^ = 84%) and wound infection (OR 0.47, 95% CI 0.19 to 1.13, p = 0.09, I^2^ = 0%). The heterogeneity of length of stay increased a little. The rest of the outcomes were consistent with the overall analysis. Heterogeneity of estimated blood loss and overall complications increased, while it reduced in retrieved lymph nodes.

### RA subgroup

When only studies with robot were analyzed, it comes out that there was no statistical difference in operative time (MD 100.71 min, 95% CI −35.12 to 236.54 min, p = 0.15, I^2^ = 96%), and there was an increase in the degree of heterogeneity. There were not enough studies referred to the three grades of delayed gastric emptying, so no meta-analysis was carried out. The rest of the outcomes were consistent with the overall analysis. And except for oncologic outcomes, the heterogeneity reduced. Though the heterogeneity of positive surgical margins increased significantly (I^2^ = 51%), no difference was found when a random-effects model was used (p = 0.54).

### HQ subgroup

7 studies were considered to be of high quality by achieving a score of ≥6. Difference in operative time became nonsignificant (MD 77.03 min, 95% CI −5.14 to 159.21 min, p = 0.07, I^2^ = 95%). Complications were fewer in the MIPD group (OR 0.62, 95% CI 0.42 to 0.92, p = 0.02, I^2^ = 0%). The other variables remained similar to the original MIPD versus OPD analysis. Heterogeneity of operative time, Grade A of pancreatic fistula, length of stay, and retrieved lymph nodes increased a little, while heterogeneity of estimated blood loss reduced. Although the heterogeneity of positive surgical margins increased significantly (I^2^ = 58%), no difference was found when a random-effects model was used (p = 0.39).

### LSS subgroup

Four studies with more than 30 patients in each procedure were also compared. No statistical difference was found in operative time (MD 84.06 min, 95% CI −27.20 to 195.32 min, p = 0.14, I^2^ = 97%) and the rate of wound infection (OR 0.44, 95% CI 0.19 to 1.00, p = 0.05, I^2^ = 0%), while complications were fewer in the MIPD group (OR 0.62, 95% CI 0.42 to 0.92, p = 0.02, I^2^ = 0%). For there was only one study which mentioned Grade A&C delayed gastric emptying, no meta-analysis was carried out. Besides, the meta-analyses showed MIPD has an advantage over OPD in oncologic outcomes, including both retrieved lymph nodes (MD 4.5, 95% CI 1.12 to 7.89, p = 0.009, I^2^ = 73%) and positive surgical margins (OR 0.27, 95% CI 0.10 to 0.73, p = 0.01, I^2^ = 0%). The remaining results were similar to the original analysis. Heterogeneity of operative time and length of stay increased a little, while heterogeneity of estimated blood loss, overall complications, and oncologic outcomes reduced.

### Publication bias

**Figure 5:**
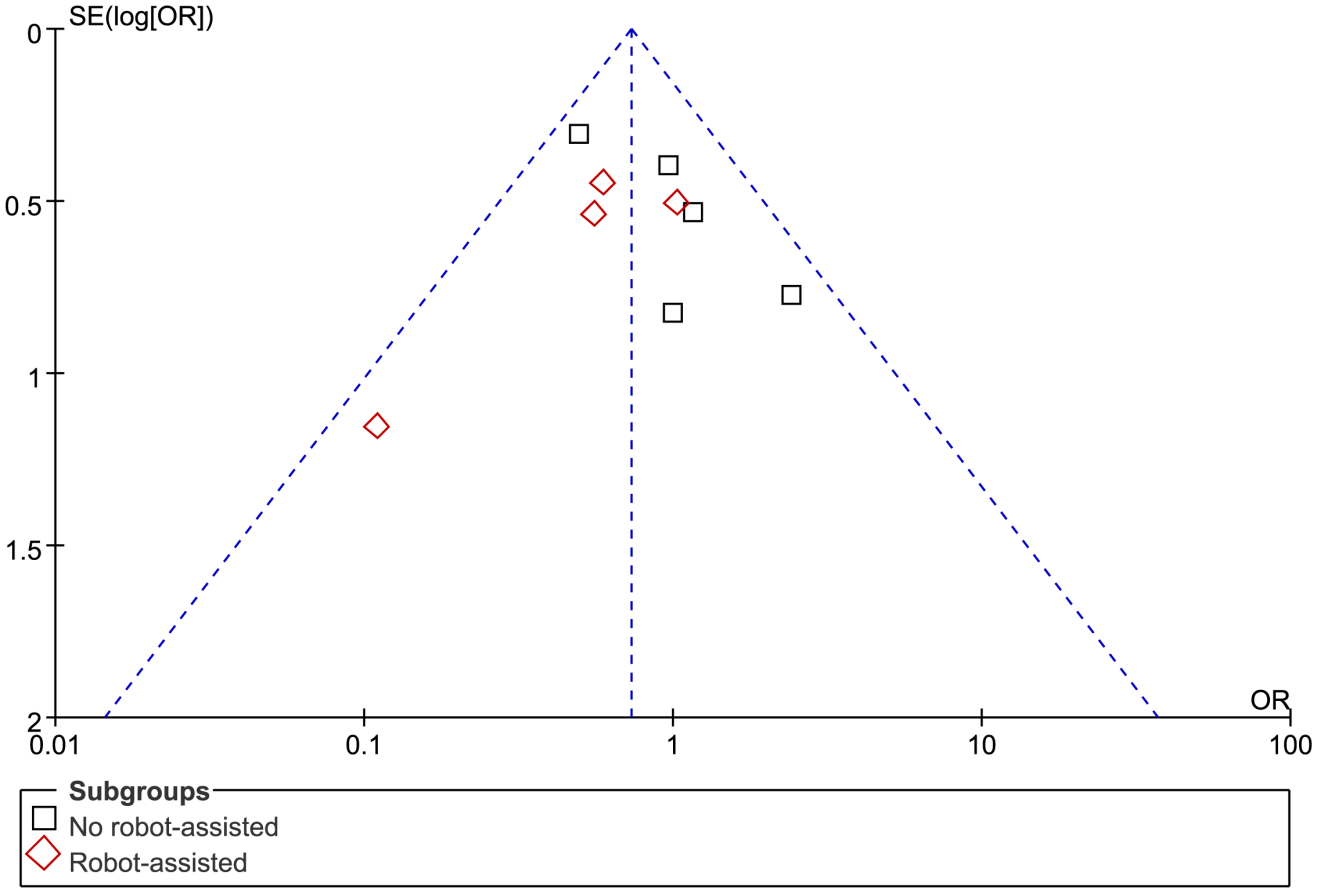
Funnel plot of overall complications in included studies, showing no publication bias.

The funnel plot of this study based on overall complications is shown in **Figure 5**. All studies lay inside the limits of the 95% CIs and distributed evenly about the vertical, showing no evidence of publication bias.

## Discussion

### Summary of Evidence

The evolution of minimally invasive approaches represents one of the most significant advances in the field of general surgery. Till now, it has proved to be beneficial to patients in terms of postoperative recovery, reduction of complications and hospital stay [1]–[5] on various occasions. With the maturation of surgeons' laparoscopic skills and advances in technology, including surgical robotics, it becomes possible in complex operations. As a new progression strategy, MIPD for pancreatic surgery is still in its early stages, and information regarding the comparison of using minimally invasive procedures over conventional OPD is rare. In the present study, a deliberate evaluation and meta-analysis using the largest available dataset of published studies were made. Gumbs AA [19] reported the rate of conversion to an open procedure was 9% after reviewing 285 published cases of MIPD. In line with it, the mean rate of the eleven studies was about 9.5%. The results demonstrated that MIPD was associated with a reduction in estimated blood loss, wound infection, and a shorter hospital stay. However, the use of minimally invasive procedures brings longer operative time. There were no significant differences between the two procedures in the likelihood of the overall complications, pancreatic fistula, delayed gastric emptying, reoperation, mortality, and oncologic outcomes. The findings of sensitivity and subgroup analysis are in most cases consistent with the pooled analysis, implying the credibility of our results.

Operative time was shown to be longer in MIPD in the pooled analysis. This is often attributed to the complexity of the procedure, including difficulties in access and exposure of the pancreas, hemorrhage control from major vasculature, particularly the reconstruction of a technically demanding reconstruction of the biliary and pancreatic remnants. But because of surgical robotics, it may make it possible to reduce the time. As expected, there was no statistical difference in the RA subgroup between MIPD&OPD, while it was statistically longer in the NRA subgroup. Considering the factor of experience, surgeons who performed more MIPD may be more skilled, the outcome from the large sample size subgroup is well explained. So we can see Buchs N C et al [36] reported that the time they spent on MIPD (444±93.5 min) was less than that on OPD (559±135 min). However, we can still find there's a trend that MIPD may cost longer operative time in all the four subgroups because the surgeons are not experienced enough.

Intraoperative blood loss was proved to be lower in the pooled analysis and all the four subgroups. This is often attributed to the magnified view afforded by laparoscopy which enhances the surgeon's view of the structures surrounding the specimen, allowing precise dissection along appropriate planes. But it also needs to be noticed that during preoperative assessment, those with expected bleeding or vascular involvement were directly decided to receive an open PD by the surgeons. It obviously led to selection bias, because these patients were expected with more blood loss. However, hemorrhage control during MIPD is extremely important or it may lead to massive bleeding. And blood loss during MIPD will obscure the view of laparoscopy, so the surgeon must perform a more careful dissection.

Overall complications should be investigated and reported out past the standard inpatient or 30-day hospitalization, which all the included studies didn't follow. Besides, it's hard for clinicians to observe and record all complications by the same criteria after such a complex operation. What's more, patients' characteristics, especially their ASA score, chronic pancreatitis and pancreatic trauma, have high influences on postoperative complications, but they were not well matched in these studies. Therefore, the real result of overall complications needs more data or studies to be evaluated.

Considering no RCT was available and information about definitions of wound infection, as well as antibiotics used in the perioperative period were not mentioned in most of the eleven studies, this question remains to be research.

The most severe complications of pancreaticoduodenectomy are pancreatic fistula and delayed gastric emptying, which have a close relationship with postoperative recovery and mortality. Our meta-analyses showed there were no statistical differences in neither overall pancreatic fistula and its three grades nor delayed gastric emptying and its three grades. And there was no difference in mortality and rate of reoperation. These indeed proved the safety of MIPD. However, considering definitions of PF&DGE were not mentioned in some included studies (**Table 1**) and most of the cases didn't mention the softness of the pancreas and the diameter of the main pancreatic duct, which are tightly related to PF [40], [41], there might be flaws in our analyses.

Our meta-analysis showed MIPD has a shorter length of stay. But it varies a lot inside the MIPD group as well as inside the OPD group. We think it may have been related to local preferences and different health care systems. A short hospital stay theoretically can decrease the pain of the patients and cost of the whole treatment, and therefore brings lots of benefits.

Considering that patients received pancreaticoduodenectomy were in most cases because of malignant tumors, comparison of oncologic safety between MIPD and OPD became important. In our pooled and sensitivity analyses, oncologic outcomes were not statistically different except in the large sample size subgroup, which showed either the number of retrieved lymph nodes or rate of R0 resection, was better in MIPD. But either the pooled or sensitive studies showed the trend that MIPD had better oncologic outcomes. However, the number of retrieved lymph nodes and rate of R0 resection can just speculate it indirectly; besides, long-term oncologic outcomes were not addressed in these referred studies. We need more long-term researches to answer this question.

### Limitations

The strength of this review lies in that it provides a comprehensive comparison of MIPD with OPD. To our knowledge, this meta-analysis is the first to expound this important issue in different subgroups. This meta-analysis has some limitations that must be taken into account. The strengths and limitations of meta-analytical techniques have been a source of considerable debate. Besides, there were no RCT contained in the meta-analysis, and the calculated results from such trials may have many biases. For example, surgeons may avoid using laparoscopy on patients with larger and more advanced tumors, and most studies didn't mention how control groups were selected. So the included patients were not well matched in age, gender, BMI, ASA scores and so on. But as RCT similar to this meta-analysis is unattainable because of ethical problems, the outcomes from non-RCT would be valuable. Furthermore, we have no convincing evidence that the number of lymph nodes retrieved during a pancreaticoduodenectomy is associated with significant end-point outcome and in particular long term survival. So we need more researches in this field. Still, only one study [30] mentioned the cost and it showed LPD was associated with equivalent overall cost compared with OPD. Obviously there should be more studies to prove it. Ultimately, the analysis included only eleven studies. The sample size of this study was not large enough, and the results require confirmation in further high-quality trials.

## Conclusions

Because of the advantages in intraoperative blood loss, wound complications, and length of stay, MIPD is worthwhile. However, considering the selection bias, the complexity of MIPD and lack of long-term oncologic outcomes, we suggest it be performed in a high-volume pancreatic surgery center in patients with light pancreatitis or small cancers distant from the major vessels. Further studies which control for biases and discuss the benefits and negatives of these procedures are needed.

## Supporting Information

Checklist S1 **PRISMA checklist.**
(DOC)

## References

[1] Qiu J, Pankaj P, Jiang H, Zeng Y, Wu H (2013) Laparoscopy versus open distal gastrectomy for advanced gastric cancer: a systematic review and meta-analysis. Surg Laparosc Endosc Percutan Tech 23: 1–7. 23386142 10.1097/SLE.0b013e3182747af7

[2] Barlehner E, Anders S, Schwetling R (2002) Laparoscopic resection of the left pancreas: technique and indication. Dig Surg 19: 507–510. 12499746 10.1159/000067606

[3] Qiu JG, Wu H, Jiang H, Huang JW, Pankaj P, et al (2011) Laparoscopic fenestration vs open fenestration in patients with congenital hepatic cysts: a meta-analysis. World J Gastroenterol 17: 3359–3365. 21876626 10.3748/wjg.v17.i28.3359 PMC3160542

[4] Huscher CG, Mingoli A, Sgarzini G, Sansonetti A, Di Paola M, et al (2005) Laparoscopic versus open subtotal gastrectomy for distal gastric cancer: five-year results of a randomized prospective trial. Ann Surg 241: 232–237. 15650632 10.1097/01.sla.0000151892.35922.f2 PMC1356907

[5] Qiu J, Chen S, Prasoon P, Wu H (2013) Meta-analysis of laparoscopic versus open distal pancreatectomy for pancreatic diseases. Surgical Practice 17: 49–57.

[6] Palanivelu C, Rajan PS, Rangarajan M, Vaithiswaran V, Senthilnathan P, et al (2009) Evolution in techniques of laparoscopic pancreaticoduodenectomy: a decade long experience from a tertiary center. J Hepatobiliary Pancreat Surg 16: 731–740. 19652900 10.1007/s00534-009-0157-8

[7] Dulucq JL, Wintringer P, Mahajna A (2006) Laparoscopic pancreaticoduodenectomy for benign and malignant diseases. Surg Endosc 20: 1045–1050. 16736311 10.1007/s00464-005-0474-1

[8] Zheng MH, Feng B, Lu AG, Li JW, Hu WG, et al (2006) Laparoscopic pancreaticoduodenectomy for ductal adenocarcinoma of common bile duct: a case report and literature review. Med Sci Monit 12: CS57–60. 16733489

[9] Palanivelu C, Jani K, Senthilnathan P, Parthasarathi R, Rajapandian S, et al (2007) Laparoscopic pancreaticoduodenectomy: technique and outcomes. J Am Coll Surg 205: 222–230. 17660068 10.1016/j.jamcollsurg.2007.04.004

[10] Pugliese R, Scandroglio I, Sansonna F, Maggioni D, Costanzi A, et al (2008) Laparoscopic pancreaticoduodenectomy: a retrospective review of 19 cases. Surg Laparosc Endosc Percutan Tech 18: 13–18. 18287976 10.1097/SLE.0b013e3181581609

[11] Tacconelli E (2010) Systematic reviews: CRD's guidance for undertaking reviews in health care. The Lancet Infectious Diseases 10: 226.

[12] Higgins JP, Green S, Collaboration C (2008) Cochrane handbook for systematic reviews of interventions: Wiley Online Library.

[13] Moher D, Liberati A, Tetzlaff J, Altman DG (2009) Group P (2009) Preferred reporting items for systematic reviews and meta-analyses: the PRISMA statement. BMJ 339: b2535. 19622551 10.1136/bmj.b2535 PMC2714657

[14] Athanasiou T, Al-Ruzzeh S, Kumar P, Crossman M-C, Amrani M, et al (2004) Off-pump myocardial revascularization is associated with less incidence of stroke in elderly patients. The Annals of thoracic surgery 77: 745–753. 14759484 10.1016/j.athoracsur.2003.07.002

[15] Clarke M, Horton R (2001) Bringing it all together: Lancet-Cochrane collaborate on systematic reviews. The Lancet 357: 1728. 10.1016/S0140-6736(00)04934-5 11403806

[16] Stroup DF, Berlin JA, Morton SC, Olkin I, Williamson GD, et al (2000) Meta-analysis of observational studies in epidemiology: a proposal for reporting. Meta-analysis Of Observational Studies in Epidemiology (MOOSE) group. JAMA 283: 2008–2012. 10789670 10.1001/jama.283.15.2008

[17] Higgins JP, Thompson SG, Deeks JJ, Altman DG (2003) Measuring inconsistency in meta-analyses. BMJ 327: 557–560. 12958120 10.1136/bmj.327.7414.557 PMC192859

[18] Farid S, Morris-Stiff G (2013) Laparoscopic vs open pancreaticoduodenectomy. J Am Coll Surg 216: 1220–1221. 23683780 10.1016/j.jamcollsurg.2013.02.018

[19] Gumbs AA, Rodriguez Rivera AM, Milone L, Hoffman JP (2011) Laparoscopic pancreatoduodenectomy: a review of 285 published cases. Ann Surg Oncol 18: 1335–1341. 21207166 10.1245/s10434-010-1503-4

[20] Lai EC, Tang CN (2013) Current status of robot-assisted laparoscopic pancreaticoduodenectomy and distal pancreatectomy: a comprehensive review. Asian J Endosc Surg 6: 158–164. 23710970 10.1111/ases.12040

[21] Kendrick ML, Cusati D (2010) Total laparoscopic pancreaticoduodenectomy: feasibility and outcome in an early experience. Arch Surg 145: 19–23. 20083750 10.1001/archsurg.2009.243

[22] Underwood RA, Soper NJ (1999) Current status of laparoscopic surgery of the pancreas. J Hepatobiliary Pancreat Surg 6: 154–164. 10398903 10.1007/s005340050099

[23] Nakamura M, Nakashima H (2013) Laparoscopic distal pancreatectomy and pancreatoduodenectomy: is it worthwhile? A meta-analysis of laparoscopic pancreatectomy. J Hepatobiliary Pancreat Sci 20: 421–428. 23224732 10.1007/s00534-012-0578-7

[24] Gumbs AA, Gres P, Madureira FA, Gayet B (2008) Laparoscopic vs. open resection of noninvasive intraductal pancreatic mucinous neoplasms. J Gastrointest Surg 12: 707–712. 17909923 10.1007/s11605-007-0311-z

[25] Dulucq JL, Wintringer P, Stabilini C, Feryn T, Perissat J, et al (2005) Are major laparoscopic pancreatic resections worthwhile? A prospective study of 32 patients in a single institution. Surg Endosc 19: 1028–1034. 16027987 10.1007/s00464-004-2182-7

[26] Kuroki T, Adachi T, Okamoto T, Kanematsu T (2012) A non-randomized comparative study of laparoscopy-assisted pancreaticoduodenectomy and open pancreaticoduodenectomy. Hepato-gastroenterology 59: 570–573. 21940382 10.5754/hge11351

[27] Zureikat AH, Breaux JA, Steel JL, Hughes SJ (2011) Can laparoscopic pancreaticoduodenectomy be safely implemented? J Gastrointest Surg 15: 1151–1157. 21538192 10.1007/s11605-011-1530-x

[28] Cho A, Yamamoto H, Nagata M, Takiguchi N, Shimada H, et al (2009) Comparison of laparoscopy-assisted and open pylorus-preserving pancreaticoduodenectomy for periampullary disease. Am J Surg 198: 445–449. 19342003 10.1016/j.amjsurg.2008.12.025

[29] Asbun HJ, Stauffer JA (2012) Laparoscopic vs open pancreaticoduodenectomy: overall outcomes and severity of complications using the Accordion Severity Grading System. J Am Coll Surg 215: 810–819. 22999327 10.1016/j.jamcollsurg.2012.08.006

[30] Mesleh MG, Stauffer JA, Bowers SP, Asbun HJ (2013) Cost analysis of open and laparoscopic pancreaticoduodenectomy: a single institution comparison. Surg Endosc 27: 4518–4523. 23943116 10.1007/s00464-013-3101-6

[31] Li Y, Wang X, Wang M, Yang Z, Peng B (2013) [Delayed gastric emptying after laparoscopic versus open pancreaticoduodenectomy: a comparative study]. Zhonghua wai ke za zhi [Chinese journal of surgery] 51: 304–307. 23895749

[32] Chalikonda S, Aguilar-Saavedra JR, Walsh RM (2012) Laparoscopic robotic-assisted pancreaticoduodenectomy: a case-matched comparison with open resection. Surg Endosc 26: 2397–2402. 22437947 10.1007/s00464-012-2207-6

[33] Zhou NX, Chen JZ, Liu Q, Zhang X, Wang Z, et al (2011) Outcomes of pancreatoduodenectomy with robotic surgery versus open surgery. Int J Med Robot 7: 131–137. 21412963 10.1002/rcs.380

[34] Bao PQ, Mazirka PO, Watkins KT (2013) Retrospective Comparison of Robot-Assisted Minimally Invasive Versus Open Pancreaticoduodenectomy for Periampullary Neoplasms. J Gastrointest Surg. 10.1007/s11605-013-2410-3 24234245

[35] Lai EC, Yang GP, Tang CN (2012) Robot-assisted laparoscopic pancreaticoduodenectomy versus open pancreaticoduodenectomy—a comparative study. Int J Surg 10: 475–479. 22732431 10.1016/j.ijsu.2012.06.003

[36] Buchs NC, Addeo P, Bianco FM, Ayloo S, Benedetti E, et al (2011) Robotic versus open pancreaticoduodenectomy: a comparative study at a single institution. World J Surg 35: 2739–2746. 21947494 10.1007/s00268-011-1276-3

[37] Bassi C, Dervenis C, Butturini G, Fingerhut A, Yeo C, et al (2005) Postoperative pancreatic fistula: an international study group (ISGPF) definition. Surgery 138: 8–13. 16003309 10.1016/j.surg.2005.05.001

[38] Wente MN, Bassi C, Dervenis C, Fingerhut A, Gouma DJ, et al (2007) Delayed gastric emptying (DGE) after pancreatic surgery: a suggested definition by the International Study Group of Pancreatic Surgery (ISGPS). Surgery 142: 761–768. 17981197 10.1016/j.surg.2007.05.005

[39] Suc B, Msika S, Fingerhut A, Fourtanier G, Hay JM, et al (2003) Temporary fibrin glue occlusion of the main pancreatic duct in the prevention of intra-abdominal complications after pancreatic resection: prospective randomized trial. Ann Surg 237: 57–65. 12496531 10.1097/00000658-200301000-00009 PMC1513966

[40] Gouma DJ, Van Geenen RC, van Gulik TM, de Haan RJ, de Wit LT, et al (2000) Rates of complications and death after pancreaticoduodenectomy: risk factors and the impact of hospital volume. Annals of surgery 232: 786. 11088073 10.1097/00000658-200012000-00007 PMC1421271

[41] Tranchart H, Gaujoux S, Rebours V, Vullierme MP, Dokmak S, et al (2012) Preoperative CT scan helps to predict the occurrence of severe pancreatic fistula after pancreaticoduodenectomy. Ann Surg 256: 139–145. 22609844 10.1097/SLA.0b013e318256c32c

